# P03.16. Student centered learning to practice patient-centered integrative medicine: the ESPRI2T approach

**DOI:** 10.1186/1472-6882-12-S1-P269

**Published:** 2012-06-12

**Authors:** C Scheffer, D Tauschel, D Cysarz, G Lutz, M Neumann, F Edelhäuser

**Affiliations:** 1 University of Witten/Herdecke, ICURAM, Witten, Germany

## Purpose

In 2004, the Integrated Curriculum for Anthroposophic Medicine (ICURAM) was launched to educate medical students in patient-centered integrative care and to develop appropriate didactic formats for that purpose.

## Methods

A six-year program was developed with longitudinally integrated modules complementing the regular medical curriculum. The educational strategy behind the program is the ESPRI2T approach. It combines Explorative learning, Supported participation, Patient-based learning, Reflective practice, Integrated Learning, Integrative Approach and Team-based learning. Student participation was assessed based on credit points achieved per year (ctp/y) through the ICURAM (1 ctp = 25–30 hour workload). The impact of the new and innovational didactic formats was evaluated by examining those adopted for use outside the ICURAM.

## Results

Fifty-five percent of 412 medical students participated in the program: 16% full participation (>4 ctp/y), 18% partial participation (1–3.99 ctp/y) and 22% occasional participation (0.25–0.99 ctp/y). Five didactic innovations were adopted by the medical school for use in the regular medical curriculum.

## Conclusion

The ICURAM program has been widely accepted and appreciated by both medical students and the medical school. The combination of patient-centeredness and student-centeredness as in the ESPRI2T approach presents a promising means of educating students in patient-centered integrative care.

